# Unraveling the Mechanics of a Repeat-Protein Nanospring: From Folding of Individual Repeats to Fluctuations of the Superhelix

**DOI:** 10.1021/acsnano.1c09162

**Published:** 2022-03-08

**Authors:** Marie Synakewicz, Rohan S. Eapen, Albert Perez-Riba, Pamela J. E. Rowling, Daniela Bauer, Andreas Weißl, Gerhard Fischer, Marko Hyvönen, Matthias Rief, Laura S. Itzhaki, Johannes Stigler

**Affiliations:** † Department of Pharmacology, University of Cambridge, Tennis Court Road, Cambridge CB2 1PD, United Kingdom^†^; ‡ Physik-Department, Technische Universität München, James-Franck-Straße 1, 85748 Garching, Germany; § Department of Biochemistry, University of Cambridge, Tennis Court Road, Cambridge, CB2 1GA, United Kingdom; ∥ Gene Center Munich, Ludwig-Maximilians-Universität München, Feodor-Lynen-Straße 25, 81377 München, Germany

**Keywords:** repeat proteins, protein folding, Ising models, protein mechanics, optical tweezers

## Abstract

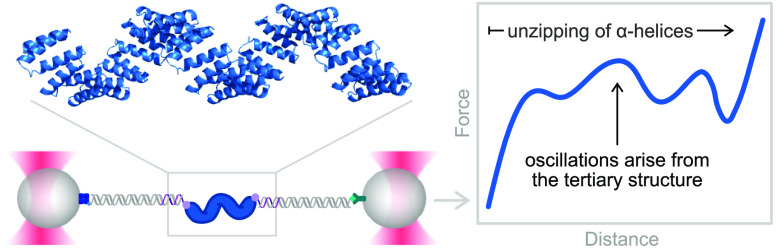

Tandem-repeat proteins comprise small secondary structure motifs that stack to form one-dimensional arrays with distinctive mechanical properties that are proposed to direct their cellular functions. Here, we use single-molecule optical tweezers to study the folding of consensus-designed tetratricopeptide repeats (CTPRs), superhelical arrays of short helix-turn-helix motifs. We find that CTPRs display a spring-like mechanical response in which individual repeats undergo rapid equilibrium fluctuations between partially folded and unfolded conformations. We rationalize the force response using Ising models and dissect the folding pathway of CTPRs under mechanical load, revealing how the repeat arrays form from the center toward both termini simultaneously. Most strikingly, we also directly observe the protein’s superhelical tertiary structure in the force signal. Using protein engineering, crystallography, and single-molecule experiments, we show that the superhelical geometry can be altered by carefully placed amino acid substitutions, and we examine how these sequence changes affect intrinsic repeat stability and inter-repeat coupling. Our findings provide the means to dissect and modulate repeat-protein stability and dynamics, which will be essential for researchers to understand the function of natural repeat proteins and to exploit artificial repeats proteins in nanotechnology and biomedical applications.

Approximately one-third of proteins in the human proteome contain repetitive motifs of varying size and structural composition.^1,2^ Within this group, members of the tandem-repeat protein class stand out due to their striking three-dimensional shapes that arise from the stacking of small secondary structure motifs of 20 to 40 amino acids into either quasi-one-dimensional arrays (solenoids) or doughnut-like shapes (toroids). Examples include tetratricopeptide, ankyrin, HEAT, and armadillo repeats. Due to their structural simplicity, repeat proteins have been recognized very early to have tremendous potential for applications in nanotechnology, *e.g.*, as synthetic biomaterials, and in biomedicine as antibody alternatives.^3,4^ Previous experiments employed ensemble biochemical techniques to study the folding of repeat proteins and to address the question of how alterations in protein sequence translate to changes in protein stability, dynamics, and structure.^5−7^ However, despite two decades of research, we still do not know how the sequence, shape, stability, and dynamics of individual repeats translates to the thermodynamic and mechanical properties of the whole array. Many all-helical repeat proteins resemble springs and have indeed been shown to be flexible molecules with spring-like properties, both of which are thought to be crucial to their biological functions.^8−18^ Despite this defining feature, we currently have a very limited understanding of the mechanics because the only methodology used to date, atomic force microscopy (AFM), lacks sensitivity in the low-pN regime relevant for these α-helical proteins. Here, we interrogate repeat-protein mechanics by using optical tweezers,^19^ which allow us to directly observe conformational transitions close to equilibrium in the low-pN range. Long-term stability and high time resolution of the instrument enable us to simultaneously manipulate single repeat proteins, study their spring-like mechanics, and obtain detailed information on their dynamics and equilibrium energetics.

Our research focuses on the tetratricopeptide repeat (TPR), which comprises a helix-turn-helix motif and is found in arrays of 3 to 16 repeats in nature^20,21^ (Figure 1A). The packing of the TPR motif results in superhelical structures^22^ (Figure 1B), which means that, of all the different repeat-protein types, TPR arrays most closely resemble a physical spring. The functions of TPR proteins are diverse, ranging from scaffolds of multiprotein assemblies regulating cell division to molecular chaperones and mediators of bacterial quorum sensing.^21,23−25^ Consensus-designed TPRs (CTPRs) are good candidates for building “made to measure” proteins, because they form stable arrays and are very amenable to manipulation including loop insertions.^26−28^ Their chemical stability has been characterized previously,^22,27,29−36^ whereas in contrast to other repeat proteins,^12,14,37−41^ their mechanical properties remain unexplored. For the above reasons, we choose CTPRs as a model system with which to determine how repeat energetics are connected to both the shape and the mechanics of the superhelix. To achieve this goal, we rationally redesign the geometry of the TPR superhelix by substituting residues at the repeat interface and then examine the energetic and mechanical response of these changes using Ising models and single-molecule force spectroscopy. Intriguingly, we find that the force response of CTPRs is very different from that of any other protein reported to date. In particular, their rapid dynamics allow them to unfold and refold at equilibrium over a large range of loading rates. We show that by using force we can access the full energy landscape of the CTPR array, which was previously impossible and which allows us to now accurately determine the effect of the chosen mutations. Collectively, our methodology and findings on CTPRs represent an important step in guiding future research directions to link repeat protein structure to function in nature.

**Figure 1:**
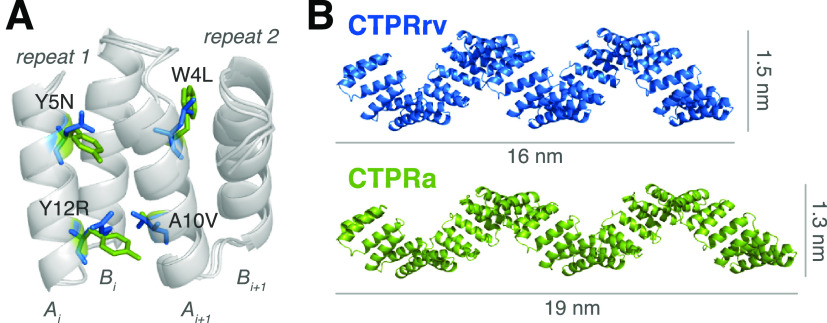
Design of the CTPRrv variant (blue) based on the original CTPRa (green; PDB accession code: 2HYZ(22)). (A) Structural representation of two neighboring CTPRs highlighting the interfacial mutations introduced in CTPRa to form CTPRrv. (B) The slight alteration in repeat packing leads to changes in the diameter and the length of the superhelix, here shown with an array length of 20 repeats.

## Results and Discussion

### Design and Structure Determination of a TPR Array with Altered Superhelical Geometry

The starting point for this study was a consensus-designed TPR protein (referred to as CTPRa) that adopts a superhelical structure within the crystal lattice.^22^ This superhelical geometry of the TPRs is established through stacking of the A-helix (A_*i*+1_ in Figure 1A) of a given repeat against both A- and B-helices of the preceding repeat (A_*i*_ and B_*i*_ in Figure 1A).^26^ The resulting angles between repeat planes then give rise to the pitch and diameter of the superhelix (Figure 1B).^13^ Interfaces between repeats are largely formed by bulky hydrophobic residues. Therefore, to create a CTPR variant with altered superhelical geometry, we identified four such interface residues on the basis of sequence conservation in different TPR families^26,42^ and substituted them with polar or aliphatic side chains (W4L, Y5N, A10 V, Y12R). This repeat variant is subsequently referred to as CTPRrv. Circular dichroism spectroscopy of the 5-repeat proteins used in this study shows that the CTPR α-helicity remains intact in the new repeat type (Figure S1).

We used X-ray crystallography to determine the structure of a construct composed of 4 repeats of the new variant and a C-terminal solvating helix, CTPRrv4, to 3.0 Å resolution (see Table S1 and Figure S2). CTPRrv4 crystallized in the *P*3_1_21 space group with two molecules per asymmetric unit. As was observed for CTPRa, the C-terminal solvating helix was not resolved due to a higher preference for end-to-end stacking between molecules in neighboring asymmetric units.^22^ The resolution was sufficient to determine the change in repeat plane angles using the C_α_ coordinates: while the twist remained almost unchanged, an increase in the curvature is compensated by a similar decrease in bending (Figure 1A, Table S2). Although these changes may at first appear insignificant in the context of a 4-repeat array, they translate into clear differences in the longer superhelical arrays, leading to a decrease in helix length and increase in helix diameter (Figure 1B). Furthermore, when compared to other CTPRs, all of which exhibit backbone RMSDs within 0.72 Å, the CTPRrv backbone differs by 1.4 Å relative to the CTPRa backbone.

### Single-Molecule Force–Distance Data Indicate Equilibrium Folding of CTPRs

**Figure 2:**
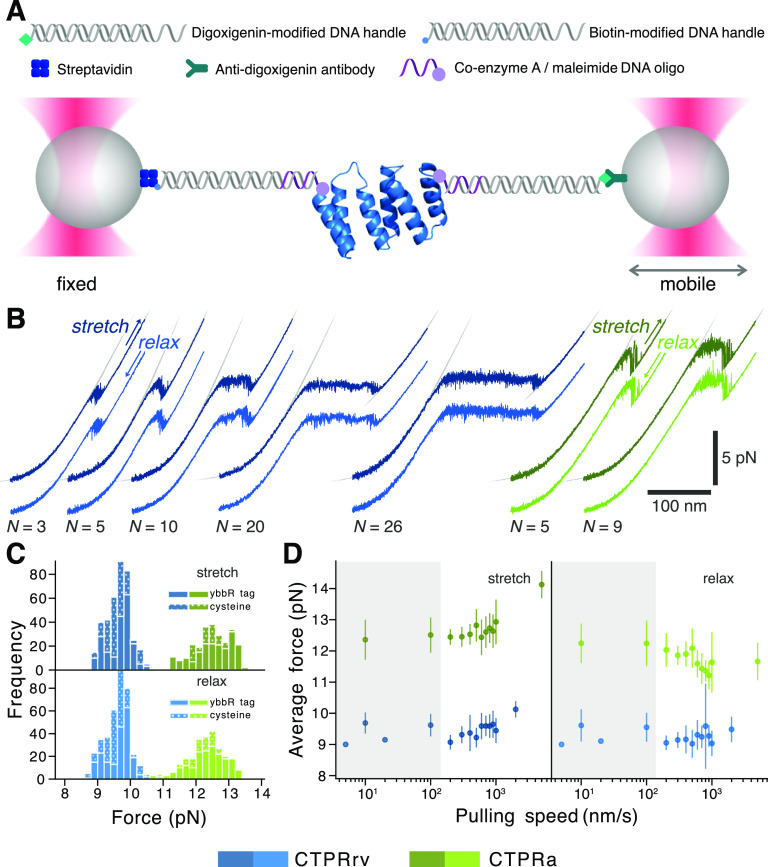
Probing the mechanics of CTPRs using optical tweezers. (A) A typical dumbbell optical tweezers setup in which the protein of interest is site-specifically tethered to dsDNA handles. A force is applied to the protein by increasing the distance between the traps. (B) Representative force–distance curves (FDCs) for CTPRrv (blue) and CTPRa (green) proteins of different array lengths pulled at 10 nm/s. The corresponding stretch and relax traces are offset for clarity as they would almost perfectly overlay otherwise. Fits for the DNA eWLC and the polypeptide–DNA construct are shown in gray, and the resulting contour lengths of the unfolded polypeptide are listed in Table S4. (C) Histograms of the plateau forces for stretch and relax FDCs of all molecules at 10 and 100 nm/s indicate that the transition remains unaffected by the attachment method. (D) The average plateau forces from stretch and relax FDCs at different pulling speeds (mean ± standard deviation) show only a modest loading rate dependence. For representative FDCs at different pulling speeds, see Figure S4. The gray shaded area highlights the low pulling speeds at which the unfolding and refolding plateau forces of both repeat types are indistinguishable, and hence, the regime in which the respective system is at equilibrium.

To examine the mechanical folding and unfolding of the CTPR superhelix, we prepared CTPRrv arrays of *N* = 3, 5, 10, 20, and 26 and CTPRa arrays of *N* = 5 and 9 for force spectroscopy measurements using optical tweezers (Figure 2A,^43^). Due to bacterial recombination, it was not possible to obtain CTPRa arrays of *N* = 10, 20, and 26 (see Section V.A.5 in the Supporting Information). Using the Sfp enzyme, the coenzyme A modified ssDNA oligos were conjugated to N- and C-terminal ybbR-tags on the protein.^44^ These protein–DNA chimeras were then attached to micron-sized silica beads using dsDNA handles.

For an initial characterization of the CTPR force response, we recorded force–distance curves (FDCs) from stretch–relax cycles at a pulling velocity of 10 and 100 nm/s (Figure 2B). The FDCs of all variants display a characteristic plateau region and a subsequent force dip, flanked by the characteristic worm-like-chain (WLC) behavior of stretching the linker, and the linker combined with the unfolded polypeptide. The plateau is preceded by a small and gradual transition that can no longer be described by a WLC. Since the plateau’s length correlates with CTPR array size, we attribute the plateau and dip regions to force-induced unfolding of CTPR repeats. The shape of the unfolding profile is indicative of sequential unfolding of repeats (plateau) until a minimally stable unit is reached, which appears to unravel in a more cooperative manner (dip). Furthermore, we noticed that FDCs from stretch and relax cycles of a single molecule at these loading rates are almost indistinguishable when superimposed. The increased noise levels of plateau and dip indicate very fast unfolding and refolding transitions (Figure 2B), which together with the absence of hysteresis, suggests that CTPR unfolding and refolding occurs at equilibrium; *i.e.*, the folding kinetics of the system under investigation are much faster than the pulling speed, allowing it to re-equilibrate instantly.

The two CTPR variants exhibit the same unfolding and refolding behavior, albeit at different forces. We estimated the plateau force of each FDC by fitting Gaussians to histograms of the respective force data (see Section VI.B. in the Supporting Information). Whereas CTPRa variants unfolded and refolded at a plateau force of ≈12.5 pN, the plateau of CRPRrv was significantly lower at ≈9.5 pN (Figure 2B,C), indicating that the introduced mutations have a destabilizing effect. Furthermore, to ensure that the intrinsic α-helicity of the ybbR-tag^45^ did not alter the stability of the repeat arrays, we compared the mechanical and chemical stability of proteins with ybbR-tags to the more commonly used cysteine modifications for maleimide-based attachment strategies (Figures 2C and S3). Although a slight stabilization was observed in chemical denaturation experiments, the ybbR-tag does not discernibly influence the unfolding or refolding plateau forces, the unfolding and refolding energies (beyond the contribution of contour length), or the character of the mechanical response (Figure 2C).

A systematic screen of pulling velocities ranging from 10 nm/s to 5 μm/s revealed that, for both CTPRrv and CTPRa, the average folding and unfolding plateau forces are only marginally affected by the loading rate, leading to a slight increase in hysteresis at pulling speeds of >500 nm/s (Figures 2D and S4). This effect is more pronounced in CTPRa arrays than in CTPRrv arrays. It is interesting to note that even at higher pulling speeds not all stretch–relax cycles exhibit hysteresis, leading to a large variation within even a single molecule (*e.g.*, see FDCs collected at 1 μm/s in Figure S4). However, most importantly, we found that there was no significant hysteresis between FDCs from stretch and relax cycles at pulling speeds ≤100 nm/s in both repeat types. The absence of a pulling speed-dependent folding/unfolding force is again evidence for rapid equilibrium fluctuations of CTPR subunits in this loading rate regime.

### Averaged Force–Distance Data Hint at the Folding Mechanism of the CTPR Arrays

**Figure 3:**
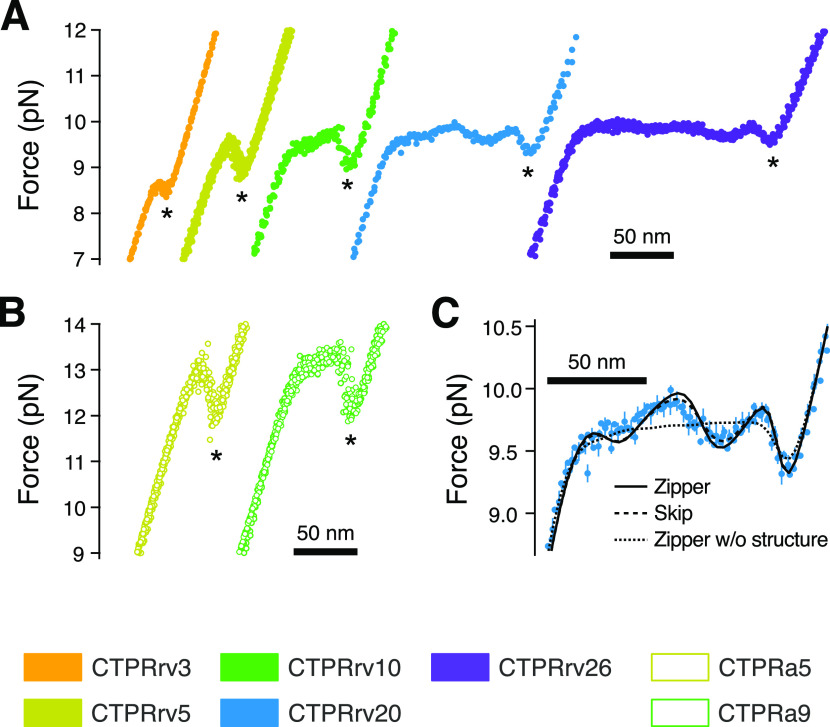
Description of the average force response of CTPRs using Ising models. (A, B) Overlays of aligned equilibrium force–distance curves (FDCs) of CTPRrv and CTPRa variants in which asterisks mark the characteristic force dip after the plateau. (C) Overlay of CTPRrv20 FDCs (cyan) together with fits to the heteropolymer helix Ising models with zipper approximation (solid line), skip approximation (dashed line), and zipper approximation without taking into account structural parameters of the superhelix (dotted line). In all overlays, average curves of three representative molecules are shown.

To obtain a more detailed picture of the underlying patters in the equilibrium force response of CTPRs, we binned FDCs of repeated stretch and relax cycles at pulling speeds ≤100 nm/s for each individual molecule to obtain one equilibrium FDC per measured molecule. Figure 3A,B shows FDCs of three molecules of each repeat type at the chosen array lengths, overlaid and aligned along force and distance coordinates to avoid the introduction of common instrumental artifacts such as miscalibration of the trap stiffness or the zero distance point. We found the plateau region was not uniformly flat after averaging, as would be expected from other related phenomena such as unconstrained DNA overstretching^46^ or the force response of the myosin coiled-coil;^47^ but rather, it showed highly reproducible force oscillations. This pattern was the clearest in the longer arrays of CTPRrv20 and CTPRrv26, which exhibit about 2 and 3 periods, respectively. Therefore, we reasoned that these oscillations arise directly from the structure of the superhelix. In contrast, the characteristic force dip at the end of each FDC was present in all arrays, and we hypothesize that it corresponds to the unfolding of a final stable unit.

### Force Response of CTPRs Can Be Modeled Using Mechanical Ising Analysis

We next set out to model the phenomena observed in our data based on thermodynamic first principles.^48−52^ For a given trap distance, the total free energy stored in the system is the sum of the folding free energy of the protein and the mechanical energy necessary to stretch the polymer linker as well as the traps. When appropriate models are used for the protein, linker, and traps, theoretical FDCs can be calculated for each CTPR, which can then be used for fitting experimental data. For a detailed description of model development, we refer the reader to Section VII in the Supporting Information.

The linear stacking of repetitive motifs has led our group and others to use Ising models to describe the folding energy of repeat proteins.^22,29,32−36,53−60^ In general, the total energy of an Ising-like system is described by a linear addition of the energies of the individual units and the interaction energies between nearest neighbors.^61,62^ In repeat proteins, the intrinsic energy of the repeating motif (Δ*G*_unit_), which can be a single helix or a whole repeat, is considered to be zero when it is unfolded and nonzero when it is folded. The interaction between two neighboring units (coupling or interfacial stability, Δ*G*_nn_) then depends on the states they are in: it is only nonzero when two neighboring repeats are folded but zero when at least one of the neighbors is unfolded.

We tested several models that differed in (i) how the smallest independent unit was defined (*repeat vs helix*), (ii) whether neighboring units were described using the same intrinsic energies (*homopolymer vs heteropolymer*), and (iii) whether or not both nearest neighbor and next-nearest neighbor interactions were taken into account (see Section VII in the Supporting Information). The homopolymer repeat and the homopolymer helix models had to be rejected as they did not fully reproduce all features observed in the experimental FDCs (Figure S6A). However, the heteropolymer helix model was able to describe all data, and energies obtained were independent of whether A- and B-helices could only interact with their nearest neighbor or could also form additional, next-nearest neighbor interactions to A- and B-helices in adjacent repeats (Figures 1A and Table S4). On the basis of the Akaike information criterion (Figure S6C), we proceeded with the heteropolymer helix model without a next-nearest neighbor interaction to avoid overparametrization.

All of the above models are computationally too expensive to be applied to CTPRrv20 and CTPRrv26 data, and hence, we considered two simplifications to reduce the conformational space. In the “skip” approximation, all configurations in which one or two folded helices are neighbored by unfolded helices on either side are eliminated. This allowed us to fit CTPRrv20 data but was still computationally too expensive to fit CTPRrv26 data. However, in the “zipper” approximation, in which unfolding occurs from the end(s), the conformational ensemble is reduced sufficiently enough to fit the data of the CTPRrv26 arrays. Notably, the modeled FDCs based on these approximation differed only marginally from the data to which both approximations could be fitted (*e.g.*, see dashed and continuous lines in Figures 3C and S7). Furthermore, the resulting energies of the “skip” and “zipper” approximations agreed within error (Table 1), and therefore, unless stated otherwise, we used the zipper approximation for all reported values.

**Table 1:** Quantitative Energetic Description of CTPRs^a^

		equilibrium denaturation^b^			area	heteropolymer Ising model (zipper approximation)			heteropolymer Ising model (skip approximation)		
type	*N*	Δ*G*_tot_	Δ*G*_unit_	Δ*G*_nn_	*W*_F_^c^	Δ*G*_tot_	Δ*G*_unit_	Δ*G*_nn_	Δ*G*_tot_	Δ*G*_unit_	Δ*G*_nn_
rv	3	–13.0 ± 0.3			–22.7 ± 0.3	–18.4 ± 0.9	1 ± 1	–10 ± 2	–18 ± 1	1.1 ± 1.1	–11 ± 2
	5	–26.1 ± 0.5			–43.4 ± 0.2	–39.7 ± 0.4	1.5 ± 0.3	–11.8 ± 0.3	–39.7 ± 0.4	1.5 ± 0.3	–11.8 ± 0.3
	10	–59 ± 1			–91 ± 1	–87 ± 3	1.2 ± 0.3	–11.0 ± 0.1	–87 ± 3	1.2 ± 0.3	–11.0 ± 0.1
	20	–125 ± 2			–177 ± 4	–173 ± 2	1.0 ± 0.3	–10.2 ± 0.2	–173 ± 2	1.1 ± 0.2	–10.3 ± 0.2
	26	–164 ± 3			–252.7 ± 0.6	–237 ± 2	0.5 ± 0.2	–10.0 ± 0.3	N/A^d^	N/A^d^	N/A^d^
	all^e^		0.20 ± 0.05	–6.8 ± 0.1			1.1 ± 0.2	–11.0 ± 0.2		1.3 ± 0.2	–11.3 ± 0.3
a	5	–46 ± 1			–63.7 ± 0.4	–61.3 ± 0.6	–2.4 ± 0.4	–12.4 ± 0.4	–61.4 ± 0.6	–2.4 ± 0.4	–12.4 ± 0.4
	9	–91 ± 2			–122.2 ± 0.9	–118 ± 2	–1.3 ± 0.3	–13.3 ± 0.4	–117 ± 2	–1.3 ± 0.3	–13.2 ± 0.4
	all^e^		–1.0 ± 0.2	–10.3 ± 0.1			–1.9 ± 0.3	–12.7 ± 0.3		–1.9 ± 0.3	–12.7 ± 0.3

a All energies are reported in units of *k*_B_*T* and *N* refers to the number of repeats. For parameters derived from Ising models, Δ*G*_unit_ is the intrinsic repeat energy, Δ*G*_nn_ is the next-neighbor interaction energy, and Δ*G*_tot_ is the total energy for an N-mer (see eqs 1 and 2). Alternatively, the folding energy can be approximated as the work done by the protein, *W*_F_, from the area under the curve.

b Results of the rv-type are based on a global fit to data of arrays with *N* = 2, 4, 5, 8, and 10 repeats;^63^ see Figure S3. Values of the a-type are as reported previously.^33^ The errors shown here were propagated from the global fit.

c See eq S7. Values are reported as mean ± s.e.m. for each repeat length.

d Values for the heteropolymer model in the skip approximation could not be computed because of limited computational capacity.

e Combined value for all repeat lengths.

### Force Oscillations Are a Consequence of the Superhelical Structure

During the initial rounds of model development, we observed that none of our Ising models alone could account for the observed force oscillations in the plateau. At first, the molecular extension of the folded portion was approximated by ξ_folded_(*c*) ≈ *n*(*c*)/*N*ξ_max_, where *n*(*c*) is the number of folded subunits in conformation *c* and ξ_max_ is the end-to-end distance of the fully folded protein. However, this assumes an arrangement of subunits in the repeat array akin to beads-on-a-string. Although all models based on this assumption for the molecular extension correctly predicted the final dip, they falsely produced a flat plateau (dotted line in Figure 3C). Only when structural parameters of the superhelix were included to account for the changes in the force vector across the folded remainder of the molecule as it unfolds was it possible to reproduce these features.

### Intrinsic and Interfacial Stabilities Are Both Modulated by Mutation

**Figure 4:**
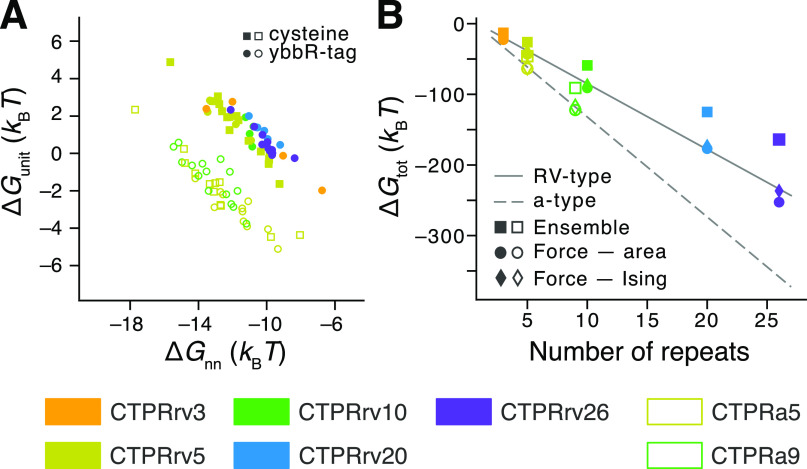
Energetic contributions to the folding free energy derived from single-molecule and ensemble measurements. (A) Intrinsic repeat energy Δ*G*_unit_ and repeat next-neighbor energy Δ*G*_nn_ for individual CTPRrv (filled symbols) and CTPRa (empty symbols) molecules based on the zipper model. For a direct comparison between results derived from skip and zipper approximations, see Figure S5. (B) Comparison of total free energies between single-molecule and ensemble measurements calculated using eq 2. The solid and dashed lines indicate simple linear regression fits to CTPRrv and CTPRa data, respectively, to guide the eye.

Using our models, we could now determine the intrinsic (Δ*G*_A_, Δ*G*_B_, Δ*G*_AB_) and interfacial contributions (Δ*G*_BA_, Δ*G*_AA_, Δ*G*_BB_) of the A- and B-helices to the free energy and from them extrapolated the repeat stability and nearest neighbor coupling with
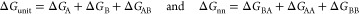
1as shown in Figure 4A and listed in Table 1. Due to the interdependence of the model parameters, the data cluster diagonally; *i.e.*, destabilization of one energetic term is compensated with stabilization of the other. However, the data of the two variants (filled *vs* empty symbols) clearly separate and are independent of attachment method (squares *vs* circles). When averaging over the whole data set, we found that the interfacial energy (Δ*G*_nn_^CTPRrv^ = −11.0 ± 0.2 *k*_B_*T*, Δ*G*_nn_^CTPRa^ = −12.7 ± 0.3 *k*_B_*T*) vastly outweighs the intrinsic energy (Δ*G*_unit_^CTPRrv^ = 1.1 ± 0.2 *k*_B_*T*, Δ*G*_unit_^CTPRa^ = −1.9 ± 0.3 *k*_B_*T*) for both repeat types. Importantly, the overall lower stability of the rv-type arrays relative to CTPRa arrays is due to a larger destabilization of the intrinsic energy than the destabilization of the interfacial energy (ΔΔ*G*_unit_ ≈ 3 *k*_B_*T* and ΔΔ*G*_nn_ ≈ 1.7 *k*_B_*T*, respectively). That is, although the chosen design did alter the helix packing between repeats more significantly than that within a repeat, the mutations not only affected both energetic parameters but also caused a rearrangement in the packing geometry that minimized destabilization of the coupling between repeats.

### The Fully Unfolded State Cannot Be Accessed in Ensemble Denaturation Experiments

Next, we determined the total free energy derived from our Ising models using
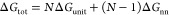
2for each variant and array length (Figure 4B, Table 1). While these values agree well with energies derived from the area under the curve of FDCs, our results show that ensemble chemical denaturation measurements substantially underestimate the total free energy of unfolding (Figures 4B and S3, Table 1). The differences between energies derived from ensemble and single-molecule force data are much larger than expected when compared to other studies in which no such discrepancy was found.^50,51,64^ A possible reason for this mismatch could arise from the formation of substantial non-native contacts in the chemically denatured ensemble. For example, Cortajarena *et al.* have shown that CTPRs can form polyproline-II (PPII) helices at high GdnHCl concentrations in which are they are likely to interact with each other.^65^ In single-molecule force experiments, the formation of PPII helices is prevented and, hence, the completely unfolded state can be accessed (as judged by the contour length, Table S5).

### CTPR Solenoids Unzip from Both Ends under Force

**Figure 5:**
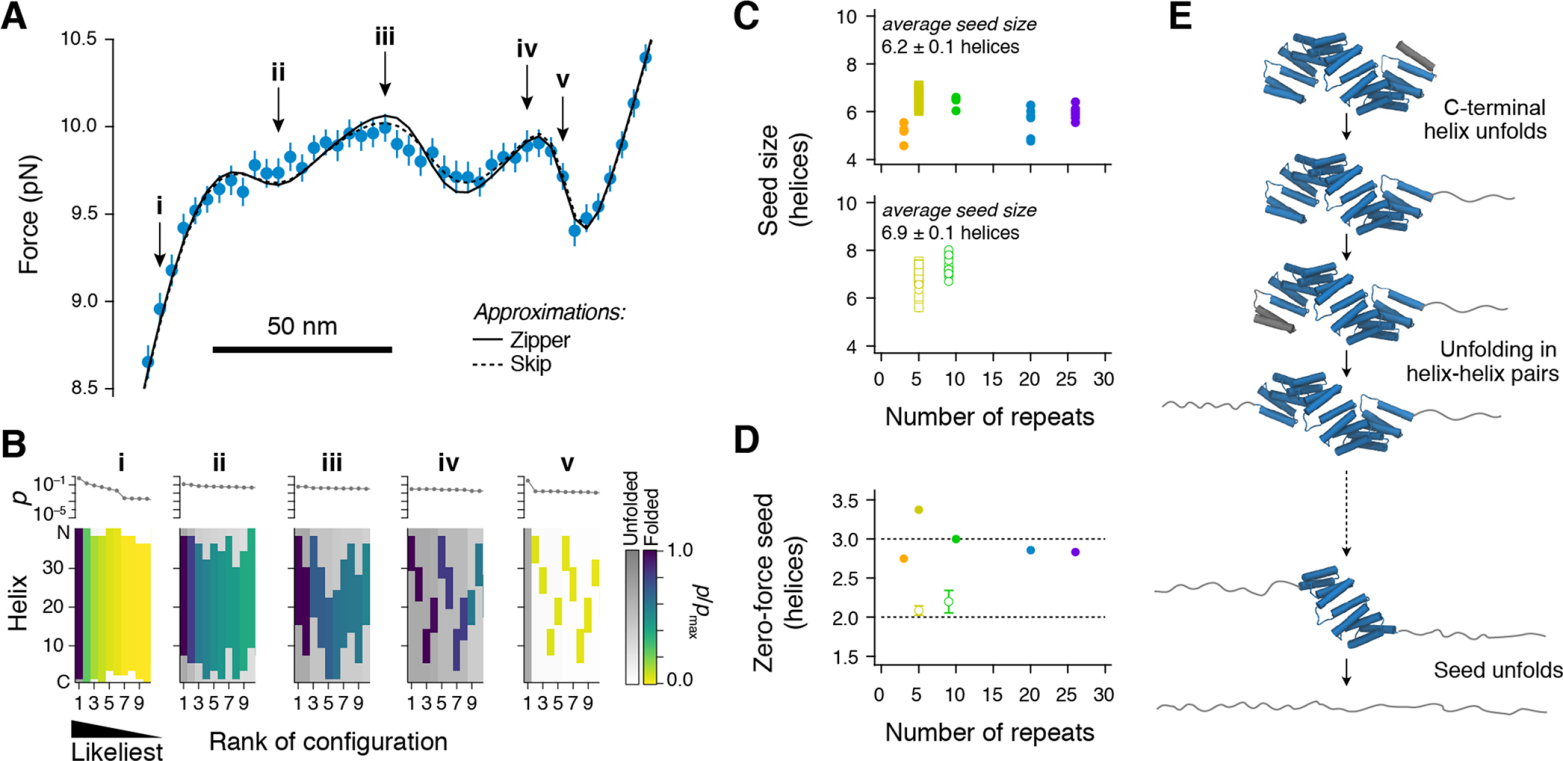
Gaining insights into the folding pathway of CTPR proteins. (A) Average force–distance data for CTPRrv20 fitted to both zipper and skip approximations of the heteropolymer helix model. Roman numerals point to distances for which snapshots of the conformational ensemble are shown in (B). (B) From the Ising model, we extract the ten likeliest configurations at each of the indicated distances in (A), ranked according to their population. Shown are the results for the skip approximation, which does *not* explicitly enforce unfolding from the ends. Colored regions in the map refer to segments of helices that are folded in a given configuration with the exact shade giving the relative probability of being folded. Gray-scale regions represent helices that are unfolded. Please note that the N–C-terminal direction is numerically reversed with the C-terminal helix having the index 0 on the *y*-axis. (C) Average size of the minimally stable folding unit in force experiments for rv- (top) and a-type repeats (bottom). Symbols and colors are the same as those in Figure 4A. (D) Inferred average minimal stable folding unit in the absence of force for rv-type (filled circles) and a-type (empty circles) variants. Error bars show the standard error of the mean. (E) A model for the force-induced unfolding of CTPRs.

On the basis of our Ising analyses, we were able to develop a model of the likeliest unfolding (and folding) pathway for CTPR proteins under mechanical load. To this end, we chose to examine the results of the skip approximation in more detail to avoid possible bias introduced by the assumption that arrays unfold from the end as it is done in the zipper approximation. For each trap distance, we recorded the likeliest conformations ranked by their population. Examples at six different distances along the average unfolding profile of CTPRrv20 are shown in Figure 5 (and Figure S7 for CTPRrv26), along with the probabilities of a given configuration and the relative probabilities of individual helices being folded or unfolded. Our data show that unfolding preferentially occurs from the ends in a zipper-like fashion. However, at increasing distances, there are several, almost equally likely conformations that have different segments of folded and unfolded helices. When plotting the probability of being folded for all helices as a function of distance (Figure S8), we further noticed that unfolding starts with the most C-terminal helix and then propagates one or two helices at a time from the C-terminus (*i.e.*, A- and B-helices alternating or almost in pairs of B_*i*–1_A_*i*_) and one repeat at a time from the N-terminus (*i.e.*, in pairs of A_*i*_B_*i*_).

The very fast equilibrium fluctuations of the C-terminal helix, and possibly also the N-terminal repeat, are beyond the time resolution of our instrument, and hence, the transition from DNA stretching into the plateau is rather smooth (*i.e.*, “averaged”), particularly in FDCs of the rv-type arrays. The asymmetry of the unfolding pathway arises directly from the structure: (i) the C-terminal B-helix is exposed without any interactions beyond those with the corresponding A-helix of the last repeat of the folded protein/remainder; (ii) the force vector aligns the molecule such that the C-terminal helix can get “un-zipped”, while at the N-terminus, a whole repeat experiences shear forces; (iii) a B_*i*–1_A_*i*_ repeat is structurally different from an A_*i*_B_*i*_ repeat, leading to different unfolding patterns from either end as the protein is unzipped by force (Figure S8). This directionality is a natural consequence of the array geometry itself, since repeats at the center of the array are less likely to unfold than those at the termini, but it is also consistent with hydrogen–deuterium exchange experiments of CTPRs^27,66^ and further studies of other designed and natural repeat proteins. For example, consensus ankyrin repeats were shown to unfold from both ends using chemical denaturation^58^ and from one end to the other under force.^39^ In contrast, some natural repeat proteins evolved to have repeats (or repeat domains) of significantly different stability, the weakest of which unfold first even if they are located at the center of the array.^67−69^

### To Seed or Not to Seed?

Many globular and natural repeat proteins first form a folding nucleus which then “seeds” the folding of the rest of the structure. In consensus repeat proteins, such a seed could potentially form anywhere along the unfolded polypeptide chain, and multiple seeds could potentially form at the same time if the polypeptide was long enough.^7^ We hypothesized that the characteristic “dip” feature at the end of the plateau region contains such a seed and expect that it is caused by the cooperative folding/unfolding of a well-defined minimally stable unit of a certain length that exchanges in a two-state manner with the unfolded state. However, our model indicates that the dip (Figure 5A,B) represents interconversions between a large ensemble of marginally stable conformations of varying size between one another and the unfolded conformation. For example, at position (v) in Figure 5A, the likeliest conformation is the unfolded state. However, this state exchanges rapidly with an ensemble of conformations with ≈6 to 7 consecutively folded helices that individually are less populated than the unfolded state but together amount to about 50% of the conformational space (Figure 5Bv). Consequently, although it is very unlikely that multiple seeds can fold under force, the definition of a seed becomes blurred in the context of CTPRs.

We then calculated the average number of folded helices once the fully unfolded conformation reaches a likelihood of 50%. As expected, the size of this seed was independent of array length and comprised approximately 6.2 ± 0.1 and 6.9 ± 0.1 helices for the rv- and a-type arrays, respectively (Figure 5C), which agrees with estimates of the contour length increase extracted from the raw data (Figure S9). However, it is important to stress that these seeds do not cooperatively exchange with the unfolded conformation in a two-state manner nor should they be mistaken for the minimal folded unit under zero force such as one would obtain in ensemble measurements. To obtain an estimate of the minimal folding unit at zero force, we instead calculated the minimal number of consecutive folded helices needed in order to achieve a negative Δ*G*_tot_ using eq 2, which were independent of variant length and were close to 3 helices for CTPRrv and 2 helices for CTPRa (Figure 5D). Hence, a single CTPRa repeat is weakly stable (−1.9 ± 0.3 *k*_B_*T*) and may fold transiently on its own. On the contrary, a single CTPRrv repeat is unstable (1.1 ± 0.2 *k*_B_*T*) and requires the energy from the interface with at least one more helix to fold.

The average “seed” size of 6 to 7 helices is, intriguingly, consistent with a folding correlation length of roughly 3 repeats proposed by coarse-grained simulations^7^ as well as a folding “nucleus” of 2.5 repeats as concluded from ensemble folding studies of a set of CTPR proteins.^32^ In contrast, our estimate of the zero-force seed is much smaller as it only refers to the number of repeating units required for Δ*G* < 0, which we would like to point out, does not mean that such a structure is fully folded at all times (*e.g.*, a single CTPRa repeat is still 13% unfolded).

### The Absence of Sawtooth-Like Unfolding Is a Consequence of Tether Elasticity

To our initial surprise, we did not observe the repeat-by-repeat, sawtooth unfolding patterns observed for other solenoid repeat proteins, particularly ankyrins.^12,14,37−40^ However, using our mechanical Ising model, we increased the effective spring constant of the system connected to the protein (optical trap and linker molecules) to simulate the much stiffer compliance of the surface and cantilever in the AFM experiments. This modification allowed us to reproduce the characteristic sawtooth pattern of the repeats unfolding one at a time for a consensus ankyrin-repeat protein with five repeats (Figure S10), highlighting that this behavior is, at least in part, related to the stiffness of the experimental apparatus rather than an intrinsic characteristic of the protein. These findings raise important questions for future research of repeat proteins under load both *in vitro* and *in vivo* as the context of the setup or the cellular environment may change how we perceive the force response of the protein of interest.

### Force Response of the Superhelical Tertiary Structure

Previously, several groups used steered MD simulations of natural repeat proteins to show that structural rearrangements at interfaces between repeats allowed the array to stretch as a whole before breaking of the array and unfolding of smaller structural elements occurred at higher forces.^10,11,16^ However, at this time, we do not have evidence that the CTPR superhelix stretches before unfolding starts at the ends or that such a response is of similar compliance to DNA and could therefore be hidden in the linker response. Instead, we found that the DNA parameters compensate for the dimension of the folded construct and can estimate the end-to-end distances prior to unfolding using a linear regression to obtain values that agree well with our structural data (Figure S11). Given that we can clearly “see” the superhelix in the force plateau, we surmise that the interfaces in the CTPR arrays are coupled too strongly to rearrange before unzipping of the terminal helices occurs. This conclusion is supported by previous findings that describe packing between CTPRs as rather rigid^70^ and that show CTPR arrays to have a spring constant much larger than that of our instrument.^8^ It remains to be seen how the balance of the intrinsic repeat stability and interfacial coupling translates into the overall stiffness of the tertiary structure to explain the flexibility of several natural repeat proteins observed in both simulations and experiments.

## Conclusion

In summary, we have resolved a truly unique force response of a solenoid repeat protein and with a sensitivity that is high enough to resolve its tertiary structure. Furthermore, using Ising models, we have shown how two geometrically distinct CTPRs can differ in their thermodynamic and mechanical properties but still retain the same overall folding profile. Our approach circumvents current drawbacks of ensemble studies, as it only requires data of a single array length and has a clearly defined unfolded state. Since repetition of small building blocks is employed across all organisms to modulate and diversify structure and function, the insights presented here give us and others a methodological basis from which to understand the biological functions of repeat proteins and to exploit them in nanotechnology and biomedicine.

## Materials and Methods

For a detailed description, see the Supporting Information Methods Section. In brief, repeat arrays were constructed in the background of a pRSET vector and expressed in *E. coli*. Equilibrium denaturation experiments were performed using guanidine hydrochloride in sodium phosphate buffer, pH 6.8, 150 mM NaCl in a 96-well plate format.^71^ CTPRrv4 with a C-terminal solvating helix was crystallized in a solution containing 0.2 M MgCl_2_, 0.1 M sodium cacodylate, pH 6.5, and 50% v/v PEG200. Further details on data collection and processing can be found in the Supporting Information Methods Section. Angles between repeat planes were calculated essentially as published previously.^13^ Constructs were prepared for force spectroscopy using site-specific modification of either terminal ybbR-tags or cysteine residues.^72,73^ All single-molecule force spectroscopy data was collected on a custom-built instrument,^74^ processed using custom scripts developed in Igor Pro (WaveMetrics), and further analyzed using Igor Pro or Python.^75−81^ Theoretical FDCs were calculated using custom C++/CUDA software. Structural representations were generated using PyMol^82^ or VMD.^83^

## Supporting Information Available

Circular dichroism data of 5-repeat arrays; crystal structures and electron densities of CTPRrv4; equilibrium denaturation data; stretch–relax cycles at varying loading rates; comparison of Ising model energies between skip and zipper approximations; model selection and Akaike information criterion; Ising models of CTPRrv26 and distance-dependent configurations, unfolding profiles, and probabilities; contour length histograms of seeds; simulated FDCs of ankyrin; DNA eWLC parameters; refinement statistics of CTPRrv4 crystals; repeat plane angles; comparison of Ising model parameters of heteropolymer and heteropolymer NNN models; detailed description of the experimental procedures (PDF)

## Accession Codes

The structure of CTPRrv4 has been deposited in the RCSB PDB with the accession code7OBI.

## Notes

Force data are available on https://github.com/StiglerLab/CTPR.

## References

[ref1] Kajava A. V. Tandem Repeats in Proteins: From Sequence to Structure. J. Struct. Biol. 2012, 179, 279–288. 10.1016/j.jsb.2011.08.009. 21884799

[ref2] Pellegrini M.; Marcotte E. M.; Yeates T. O. A Fast Algorithm for Genome-Wide Analysis of Proteins With Repeated Sequences. Proteins: Struct., Funct., Bioinf. 1999, 35, 440–446. 10.1002/(SICI)1097-0134(19990601)35:4<440::AID-PROT7>3.0.CO;2-Y. 10382671

[ref3] Romera D.; Couleaud P.; Mejias S. H.; Aires A.; Cortajarena A. L. Biomolecular Templating of Functional Hybrid Nanostructures Using Repeat Protein Scaffolds. Biochem. Soc. Trans. 2015, 43, 825–831. 10.1042/BST20150077. 26517889

[ref4] Jost C.; Plückthun A. Engineered Proteins With Desired Specificity: DARPins, Other Alternative Scaffolds and Bispecific Iggs. Curr. Opin Struct Biol. 2014, 27, 102–112. 10.1016/j.sbi.2014.05.011. 25033247

[ref5] Barrick D.; Ferreiro D. U.; Komives E. A. Folding Landscapes of Ankyrin Repeat Proteins: Experiments Meet Theory. Curr. Opin Struct Biol. 2008, 18, 27–34. 10.1016/j.sbi.2007.12.004. 18243686 PMC2680087

[ref6] Perez-Riba A.; Synakewicz M.; Itzhaki L. S. Folding Cooperativity and Allosteric Function in the Tandem-Repeat Protein Class. Philosophical Transactions of the Royal Society of London B: Biological Sciences 2018, 373, 20170188 10.1098/rstb.2017.0188. 29735741 PMC5941182

[ref7] Ferreiro D. U.; Walczak A. M.; Komives E. A.; Wolynes P. G. The Energy Landscapes of Repeat-Containing Proteins: Topology, Cooperativity, and the Folding Funnels of One-Dimensional Architectures. PloS Computational Biology 2008, 4, e1000070 10.1371/journal.pcbi.1000070. 18483553 PMC2366061

[ref8] Cohen S. S.; Riven I.; Cortajarena A. L.; De Rosa L.; D’Andrea L. D.; Regan L.; Haran G. Probing the Molecular Origin of Native-State Flexibility in Repeat Proteins. J. Am. Chem. Soc. 2015, 137, 10367–10373. 10.1021/jacs.5b06160. 26207891

[ref9] Lamboy J. A.; Kim H.; Lee K. S.; Ha T.; Komives E. A. Visualization of the Nanospring Dynamics of the IκBα Ankyrin Repeat Domain in Real Time. Proc. Natl. Acad. Sci. U. S. A. 2011, 108, 10178–10183. 10.1073/pnas.1102226108. 21628581 PMC3121830

[ref10] Grinthal A.; Adamovic I.; Weiner B.; Karplus M.; Kleckner N. Pr65, the Heat-Repeat Scaffold of Phosphatase PP2A, Is an Elastic Connector That Links Force and Catalysis. Proc. Natl. Acad. Sci. U. S. A. 2010, 107, 2467–2472. 10.1073/pnas.0914073107. 20133745 PMC2823866

[ref11] Kappel C.; Zachariae U.; Dölker N.; Grubmüller H. An Unusual Hydrophobic Core Confers Extreme Flexibility to Heat Repeat Proteins. Biophys. J. 2010, 99, 1596–1603. 10.1016/j.bpj.2010.06.032. 20816072 PMC2931736

[ref12] Kim M.; Abdi K.; Lee G.; Rabbi M.; Lee W.; Yang M.; Schofield C. J.; Bennett V.; Marszalek P. E. Fast and Forceful Refolding of Stretched α-Helical Solenoid Proteins. Biophys. J. 2010, 98, 3086–3092. 10.1016/j.bpj.2010.02.054. 20550922 PMC2884255

[ref13] Forwood J. K.; Lange A.; Zachariae U.; Marfori M.; Preast C.; Grubmüller H.; Stewart M.; Corbett A. H.; Kobe B. Quantitative Structural Analysis of Importin-β Flexibility: Paradigm for Solenoid Protein Structures. Structure 2010, 18, 1171–1183. 10.1016/j.str.2010.06.015. 20826343

[ref14] Serquera D.; Lee W.; Settanni G.; Marszalek P. E.; Paci E.; Itzhaki L. S. Mechanical Unfolding of an Ankyrin Repeat Protein. Biophys. J. 2010, 98, 1294–1301. 10.1016/j.bpj.2009.12.4287. 20371329 PMC2849098

[ref15] Baclayon M.; Ulsen P. v.; Mouhib H.; Shabestari M. H.; Verzijden T.; Abeln S.; Roos W. H.; Wuite G. J. L. Mechanical Unfolding of an Autotransporter Passenger Protein Reveals the Secretion Starting Point and Processive Transport Intermediates. ACS Nano 2016, 10, 5710–5719. 10.1021/acsnano.5b07072. 27219538

[ref16] Sotomayor M.; Corey D. P.; Schulten K. In Search of the Hair-Cell Gating Spring Elastic Properties of Ankyrin and Cadherin Repeats. Structure 2005, 13, 669–682. 10.1016/j.str.2005.03.001. 15837205

[ref17] Fukuhara N.; Fernandez E.; Ebert J.; Conti E.; Svergun D. Conformational Variability of Nucleo-Cytoplasmic Transport Factors. J. Biol. Chem. 2004, 279, 2176–2181. 10.1074/jbc.M309112200. 14561738

[ref18] Zachariae U.; Grubmüller H. Importin-β: Structural and Dynamic Determinants of a Molecular Spring. Structure 2008, 16, 906–915. 10.1016/j.str.2008.03.007. 18547523

[ref19] Žoldák G.; Rief M. Force as a Single Molecule Probe of Multidimensional Protein Energy Landscapes. Curr. Opin. Struct. Biol. 2013, 23, 48–57. 10.1016/j.sbi.2012.11.007. 23279960

[ref20] Das A. K.; Cohen P. W.; Barford D. The Structure of the Tetratricopeptide Repeats of Protein Phosphatase 5: Implications for TPR-Mediated Protein-Protein Interactions. EMBO J. 1998, 17, 1192–1199. 10.1093/emboj/17.5.1192. 9482716 PMC1170467

[ref21] D’Andrea L. D.; Regan L. TPR Proteins: the Versatile Helix. Trends Biochem. Sci. 2003, 28, 655–662. 10.1016/j.tibs.2003.10.007. 14659697

[ref22] Kajander T.; Cortajarena A. L.; Mochrie S.; Regan L. Structure and Stability of Designed TPR Protein Superhelices: Unusual Crystal Packing and Implications for Natural TPR Proteins. Acta Crystallographica Section D 2007, 63, 800–811. 10.1107/S0907444907024353. 17582171

[ref23] Do H.; Kumaraswami M. Structural Mechanisms of Peptide Recognition and Allosteric Modulation of Gene Regulation by the RRNPP Family of Quorum-Sensing Regulators. J. Mol. Biol. 2016, 428, 2793–2804. 10.1016/j.jmb.2016.05.026. 27283781 PMC4938729

[ref24] Zhang Z.; Chang L.; Yang J.; Conin N.; Kulkarni K.; Barford D. The Four Canonical TPR Subunits of Human APC/C Form Related Homo-Dimeric Structures and Stack in Parallel to Form a TPR Suprahelix. J. Mol. Biol. 2013, 425, 4236–4248. 10.1016/j.jmb.2013.04.004. 23583778 PMC3898896

[ref25] Perez-Riba A.; Itzhaki L. S. The Tetratricopeptide-Repeat Motif Is a Versatile Platform That Enables Diverse Modes of Molecular Recognition. Curr. Opin Struct Biol. 2019, 54, 43–49. 10.1016/j.sbi.2018.12.004. 30708253

[ref26] Main E. R.; Xiong Y.; Cocco M. J.; D’Andrea L.; Regan L. Design of Stable α-Helical Arrays From an Idealized TPR Motif. Structure 2003, 11, 497–508. 10.1016/S0969-2126(03)00076-5. 12737816

[ref27] Perez-Riba A.; Komives E.; Main E. R. G.; Itzhaki L. S. Decoupling a Tandem-Repeat Protein: Impact of Multiple Loop Insertions on a Modular Scaffold. Sci. Rep 2019, 9, 15439 10.1038/s41598-019-49905-4. 31659184 PMC6817815

[ref28] Diamante A.; Chaturbedy P. K.; Rowling P. J. E.; Kumita J. R.; Eapen R. S.; McLaughlin S. H.; de la Roche M.; Perez-Riba A.; Itzhaki L. S. Engineering Mono- and Multi-Valent Inhibitors on a Modular Scaffold. Chem. Sci. 2021, 12, 880–895. 10.1039/D0SC03175E. 33623657 PMC7885266

[ref29] Kajander T.; Cortajarena A. L.; Main E. R. G.; Mochrie S. G. J.; Regan L. A New Folding Paradigm for Repeat Proteins. J. Am. Chem. Soc. 2005, 127, 10188–10190. 10.1021/ja0524494. 16028928

[ref30] Cortajarena A. L.; Mochrie S. G.; Regan L. Mapping the Energy Landscape of Repeat Proteins Using NMR-Detected Hydrogen Exchange. J. Mol. Biol. 2008, 379, 617–626. 10.1016/j.jmb.2008.02.046. 18462750 PMC3282110

[ref31] Cortajarena A. L.; Regan L. Calorimetric Study of a Series of Designed Repeat Proteins: Modular Structure and Modular Folding. Protein Sci. 2011, 20, 336–340. 10.1002/pro.564. 21280125 PMC3048418

[ref32] Javadi Y.; Main E. R. G. Exploring the Folding Energy Landscape of a Series of Designed Consensus Tetratricopeptide Repeat Proteins. Proc. Natl. Acad. Sci. U. S. A. 2009, 106, 17383–17388. 10.1073/pnas.0907455106. 19805120 PMC2765091

[ref33] Perez-Riba A.; Lowe A. R.; Main E. R. G.; Itzhaki L. S. Context-Dependent Energetics of Loop Extensions in a Family of Tandem-Repeat Proteins. Biophys. J. 2018, 114, 2552–2562. 10.1016/j.bpj.2018.03.038. 29874606 PMC6129472

[ref34] Millership C.; Phillips J. J.; Main E. R. Ising Model Reprogramming of a Repeat Protein’S Equilibrium Unfolding Pathway. J. Mol. Biol. 2016, 428, 1804–1817. 10.1016/j.jmb.2016.02.022. 26947150 PMC4871810

[ref35] Cortajarena A. L.; Mochrie S. G. J.; Regan L. Modulating Repeat Protein Stability: The Effect of Individual Helix Stability on the Collective Behavior of the Ensemble. Protein Sci. 2011, 20, 1042–1047. 10.1002/pro.638. 21495096 PMC3104233

[ref36] Phillips J. J.; Javadi Y.; Millership C.; Main E. R. Modulation of the Multistate Folding of Designed TPR Proteins Through Intrinsic and Extrinsic Factors. Protein Sci. 2012, 21, 327–338. 10.1002/pro.2018. 22170589 PMC3375434

[ref37] Lee G.; Abdi K.; Jiang Y.; Michaely P.; Bennett V.; Marszalek P. E. Nanospring Behaviour of Ankyrin Repeats. Nature 2006, 440, 246–249. 10.1038/nature04437. 16415852

[ref38] Li L.; Wetzel S.; Plückthun A.; Fernandez J. M. Stepwise Unfolding of Ankyrin Repeats in a Single Protein Revealed by Atomic Force Microscopy. Biophys. J. 2006, 90, L30–L32. 10.1529/biophysj.105.078436. 16387766 PMC1367297

[ref39] Li Q.; Scholl Z. N.; Marszalek P. E. Capturing the Mechanical Unfolding Pathway of a Large Protein With Coiled-Coil Probes. Angewandte Chemie International Edition in English 2014, 53, 13429–13433. 10.1002/anie.201407211. 25339429

[ref40] Lee W.; Zeng X.; Zhou H. X.; Bennett V.; Yang W.; Marszalek P. E. Full Reconstruction of a Vectorial Protein Folding Pathway by Atomic Force Microscopy and Molecular Dynamics Simulations. J. Biol. Chem. 2010, 285, 38167–38172. 10.1074/jbc.M110.179697. 20870713 PMC2992250

[ref41] Settanni G.; Serquera D.; Marszalek P. E.; Paci E.; Itzhaki L. S. Effects of Ligand Binding on the Mechanical Properties of Ankyrin Repeat Protein Gankyrin. PLoS Comput. Biol. 2013, 9, e1002864 10.1371/journal.pcbi.1002864. 23341763 PMC3547791

[ref42] Magliery T. J.; Regan L. Beyond Consensus: Statistical Free Energies Reveal Hidden Interactions in the Design of a TPR Motif. J. Mol. Biol. 2004, 343, 731–745. 10.1016/j.jmb.2004.08.026. 15465058

[ref43] Jagannathan B.; Marqusee S. Protein Folding and Unfolding Under Force. Biopolymers 2013, 99, 860–869. 10.1002/bip.22321. 23784721 PMC4065244

[ref44] Yin J.; Lin A. J.; Golan D. E.; Walsh C. T. Site-Specific Protein Labeling by Sfp Phosphopantetheinyl Transferase. Nat. Protoc. 2006, 1, 280 10.1038/nprot.2006.43. 17406245

[ref45] Yin J.; Straight P. D.; McLoughlin S. M.; Zhou Z.; Lin A. J.; Golan D. E.; Kelleher N. L.; Kolter R.; Walsh C. T. Genetically Encoded Short Peptide Tag for Versatile Protein Labeling by Sfp Phosphopantetheinyl Transferase. Proc. Natl. Acad. Sci. U. S. A. 2005, 102, 15815–15820. 10.1073/pnas.0507705102. 16236721 PMC1276090

[ref46] van Mameren J.; Gross P.; Farge G.; Hooijman P.; Modesti M.; Falkenberg M.; Wuite G. J. L.; Peterman E. J. G. Unraveling the Structure of DNA During Overstretching by Using Multicolor, Single-Molecule Fluorescence Imaging. Proc. Natl. Acad. Sci. U. S. A. 2009, 106, 18231–18236. 10.1073/pnas.0904322106. 19841258 PMC2775282

[ref47] Schwaiger I.; Sattler C.; Hostetter D. R.; Rief M. The Myosin Coiled-Coil Is a Truly Elastic Protein Structure. Nature materials 2002, 1, 232–235. 10.1038/nmat776. 12618784

[ref48] Bockelmann U.; Essevaz-Roulet B.; Heslot F. Molecular Stick-Slip Motion Revealed by Opening DNA With Piconewton Forces. Phys. Rev. Lett. 1997, 79, 4489–4492. 10.1103/PhysRevLett.79.4489.

[ref49] Bornschlögl T.; Rief M. Single Molecule Unzipping of Coiled Coils: Sequence Resolved Stability Profiles. Phys. Rev. Lett. 2006, 96, 118102 10.1103/PhysRevLett.96.118102. 16605876

[ref50] Huguet J. M.; Bizarro C. V.; Forns N.; Smith S. B.; Bustamante C.; Ritort F. Single-Molecule Derivation of Salt Dependent Base-Pair Free Energies in DNA. Proc. Natl. Acad. Sci. U. S. A. 2010, 107, 15431–15436. 10.1073/pnas.1001454107. 20716688 PMC2932562

[ref51] Žoldák G.; Stigler J.; Pelz B.; Li H.; Rief M. Ultrafast Folding Kinetics and Cooperativity of Villin Headpiece in Single-Molecule Force Spectroscopy. Proc. Natl. Acad. Sci. U. S. A. 2013, 110, 18156 10.1073/pnas.1311495110. 24145407 PMC3831443

[ref52] Solanki A.; Neupane K.; Woodside M. T. Single-Molecule Force Spectroscopy of Rapidly Fluctuating, Marginally Stable Structures in the Intrinsically Disordered Protein α-Synuclein. Phys. Rev. Lett. 2014, 112, 158103 10.1103/PhysRevLett.112.158103. 24785077

[ref53] Mello C. C.; Barrick D. An Experimentally Determined Protein Folding Energy Landscape. Proc. Natl. Acad. Sci. U. S. A. 2004, 101, 14102–14107. 10.1073/pnas.0403386101. 15377792 PMC521126

[ref54] Marold J. D.; Kavran J. M.; Bowman G. D.; Barrick D. A Naturally Occurring Repeat Protein With High Internal Sequence Identity Defines a New Class of TPR-Like Proteins. Structure 2015, 23, 2055–2065. 10.1016/j.str.2015.07.022. 26439765 PMC4811334

[ref55] Wetzel S. K.; Settanni G.; Kenig M.; Binz H. K.; Plückthun A. Folding and Unfolding Mechanism of Highly Stable Full-Consensus Ankyrin Repeat Proteins. J. Mol. Biol. 2008, 376, 241–257. 10.1016/j.jmb.2007.11.046. 18164721

[ref56] Aksel T.; Barrick D. In Biothermodynamics, Part A; Johnson M. L., Holt J. M., Ackers G. K., Eds.; Methods in Enzymology; Academic Press: Cambridge, MA, 2009; Vol. 455; Chapter 4, pp 95–125. 10.1016/S0076-6879(08)04204-3 PMC2967780 19289204

[ref57] Aksel T.; Majumdar A.; Barrick D. The Contribution of Entropy, Enthalpy, and Hydrophobic Desolvation to Cooperativity in Repeat-Protein Folding. Structure 2011, 19, 349–360. 10.1016/j.str.2010.12.018. 21397186 PMC3151579

[ref58] Aksel T.; Barrick D. Direct Observation of Parallel Folding Pathways Revealed Using a Symmetric Repeat Protein System. Biophys. J. 2014, 107, 220–232. 10.1016/j.bpj.2014.04.058. 24988356 PMC4119276

[ref59] Geiger-Schuller K.; Barrick D. Broken Tales: Transcription Activator-Like Effectors Populate Partly Folded States. Biophys. J. 2016, 111, 2395–2403. 10.1016/j.bpj.2016.10.013. 27926841 PMC5153609

[ref60] Geiger-Schuller K.; Sforza K.; Yuhas M.; Parmeggiani F.; Baker D.; Barrick D. Extreme Stability in De Novo-Designed Repeat Arrays Is Determined by Unusually Stable Short-Range Interactions. Proc. Natl. Acad. Sci. U. S. A. 2018, 115, 7539–7544. 10.1073/pnas.1800283115. 29959204 PMC6055163

[ref61] Ising E. Beitrag Zur Theorie Des Ferromagnetismus. Zeitschrift für Physik 1925, 31, 253–258. 10.1007/BF02980577.

[ref62] Lenz W. Beitrag Zum Verständnis Der Magnetischen Erscheinungen in Festen Körpern. Physikalische Zeitschrift 1920, 21, 613–615.

[ref63] Lowe A. R.; Perez-Riba A.; Itzhaki L. S.; Main E. R. Pyfolding: Open-Source Graphing, Simulation, and Analysis of the Biophysical Properties of Proteins. Biophys. J. 2018, 114, 516–521. 10.1016/j.bpj.2017.11.3779. 29414697 PMC5985001

[ref64] Stigler J.; Rief M. Calcium-Dependent Folding of Single Calmodulin Molecules. Proc. Natl. Acad. Sci. U. S. A. 2012, 109, 17814–17819. 10.1073/pnas.1201801109. 22753517 PMC3497792

[ref65] Cortajarena A. L.; Lois G.; Sherman E.; O’Hern C. S.; Regan L.; Haran G. Non-Random-Coil Behavior as a Consequence of Extensive Ppii Structure in the Denatured State. J. Mol. Biol. 2008, 382, 203–212. 10.1016/j.jmb.2008.07.005. 18644382 PMC2603145

[ref66] Main E. R. G.; Stott K.; Jackson S. E.; Regan L. Local and Long-Range Stability in Tandemly Arrayed Tetratricopeptide Repeats. Proc. Natl. Acad. Sci. U. S. A. 2005, 102, 5721–5726. 10.1073/pnas.0404530102. 15824314 PMC556279

[ref67] Tsytlonok M.; Craig P. O.; Sivertsson E.; Serquera D.; Perrett S.; Best R. B.; Wolynes P. G.; Itzhaki L. S. Complex Energy Landscape of a Giant Repeat Protein. Structure 2013, 21, 1954–1965. 10.1016/j.str.2013.08.028. 24120762 PMC4256716

[ref68] Werbeck N. D.; Rowling P. J.; Chellamuthu V. R.; Itzhaki L. S. Shifting Transition States in the Unfolding of a Large Ankyrin Repeat Protein. Proc. Natl. Acad. Sci. U. S. A. 2008, 105, 9982–9987. 10.1073/pnas.0705300105. 18632570 PMC2481366

[ref69] Tripp K. W.; Barrick D. Rerouting the Folding Pathway of the Notch Ankyrin Domain by Reshaping the Energy Landscape. J. Am. Chem. Soc. 2008, 130, 5681–5688. 10.1021/ja0763201. 18396879 PMC2474552

[ref70] Cheng C. Y.; Jarymowycz V. A.; Cortajarena A. L.; Regan L.; Stone M. J. Repeat Motions and Backbone Flexibility in Designed Proteins With Different Numbers of Identical Consensus Tetratricopeptide Repeats. Biochemistry 2006, 45, 12175–12183. 10.1021/bi060819a. 17002317

[ref71] Perez-Riba A.; Itzhaki L. S. A Method for Rapid High-Throughput Biophysical Analysis of Proteins. Sci. Rep. 2017, 7, 9071 10.1038/s41598-017-08664-w. 28831058 PMC5567296

[ref72] Synakewicz M.; Bauer D.; Rief M.; Itzhaki L. S. Bioorthogonal Protein-DNA Conjugation Methods for Force Spectroscopy. Sci. Rep. 2019, 9, 13820 10.1038/s41598-019-49843-1. 31554828 PMC6761116

[ref73] Mukhortava A.; Schlierf M. Efficient Formation of Site-Specific Protein-DNA Hybrids Using Copper-Free Click Chemistry. Bioconjugate Chem. 2016, 27, 1559–1563. 10.1021/acs.bioconjchem.6b00120. 27322198

[ref74] von Hansen Y.; Mehlich A.; Pelz B.; Rief M.; Netz R. R. Auto- and Cross-Power Spectral Analysis of Dual Trap Optical Tweezer Experiments Using Bayesian Inference. Rev. Sci. Instrum. 2012, 83, 095116 10.1063/1.4753917. 23020428

[ref75] Millman K. J.; Aivazis M. Python for Scientists and Engineers. Computing in Science & Engineering 2011, 13, 9–12. 10.1109/MCSE.2011.36.

[ref76] Oliphant T. E. Python for Scientific Computing. Computing in Science & Engineering 2007, 9, 10–20. 10.1109/MCSE.2007.58.

[ref77] van der Walt S.; Colbert S C.; Varoquaux G. The Numpy Array: A Structure for Efficient Numerical Computation. Computing in Science & Engineering 2011, 13, 22–30. 10.1109/MCSE.2011.37.

[ref78] Hunter J. D. Matplotlib: A 2D Graphics Environment. Computing In Science & Engineering 2007, 9, 90–95. 10.1109/MCSE.2007.55.

[ref79] Waskom M.; mwaskom/seaborn: v0.11.2; 2020; https://doi.org/10.5281/zenodo.592845.

[ref80] Jones E.; Oliphant T.; Peterson P. Scipy: Open Source Scientific Tools for Python; 2001; http://www.scipy.org/.

[ref81] McKinney W. Data Structures for Statistical Computing in Python. Proceedings of the 9th Python in Science Conference 2010, 56–61. 10.25080/Majora-92bf1922-00a.

[ref82] Schrödinger, LLC Pymol Molecular Graphics System, Version 1.8; 2015; https://pymol.org/2/.

[ref83] Humphrey W.; Dalke A.; Schulten K. Vmd: Visual Molecular Dynamics. J. Mol. Graphics 1996, 14, 33–38. 10.1016/0263-7855(96)00018-5. 8744570

[ref84] Synakewicz M.; Eapen R. S.; Perez-Riba A.; Bauer D.; Weißl A.; Fischer G.; Hyvönen M.; Rief M.; Itzhaki L. S.; Stigler J. Consensus tetratricopeptide repeat proteins are complex superhelical nanosprings. bioRxiv 2021, 10.1101/2021.03.27.437344.

